# Environmental Complexity and Biodiversity: The Multi-Layered Evolutionary History of a Log-Dwelling Velvet Worm in Montane Temperate Australia

**DOI:** 10.1371/journal.pone.0084559

**Published:** 2013-12-17

**Authors:** James K. Bull, Chester J. Sands, Ryan C. Garrick, Michael G. Gardner, Noel N. Tait, David A. Briscoe, David M. Rowell, Paul Sunnucks

**Affiliations:** 1 School of Biological Sciences, Monash University, Melbourne, Victoria, Australia; 2 Natural Environment Research Council, British Antarctic Survey, Cambridge, United Kingdom; 3 Department of Biology, University of Mississippi, Oxford, Mississippi, United States of America; 4 School of Biological Sciences, Flinders University, Adelaide, South Australia, Australia; 5 Evolutionary Biology Unit, South Australian Museum, Adelaide, South Australia, Australia; 6 Department of Biological Sciences, Macquarie University, Sydney, New South Wales, Australia; 7 Research School of Biology, Australian National University, Canberra, Australian Capital Territory, Australia; University of Arkansas, United States of America

## Abstract

Phylogeographic studies provide a framework for understanding the importance of intrinsic versus extrinsic factors in shaping patterns of biodiversity through identifying past and present microevolutionary processes that contributed to lineage divergence. Here we investigate population structure and diversity of the Onychophoran (velvet worm) *Euperipatoides rowelli* in southeastern Australian montane forests that were not subject to Pleistocene glaciations, and thus likely retained more forest cover than systems under glaciation. Over a ~100 km transect of structurally-connected forest, we found marked nuclear and mitochondrial (mt) DNA genetic structuring, with spatially-localised groups. Patterns from mtDNA and nuclear data broadly corresponded with previously defined geographic regions, consistent with repeated isolation in refuges during Pleistocene climatic cycling. Nevertheless, some *E. rowelli* genetic contact zones were displaced relative to hypothesized influential landscape structures, implying more recent processes overlying impacts of past environmental history. Major impacts at different timescales were seen in the phylogenetic relationships among mtDNA sequences, which matched geographic relationships and nuclear data only at recent timescales, indicating historical gene flow and/or incomplete lineage sorting. Five major *E. rowelli* phylogeographic groups were identified, showing substantial but incomplete reproductive isolation despite continuous habitat. Regional distinctiveness, in the face of lineages abutting within forest habitat, could indicate pre- and/or postzygotic gene flow limitation. A potentially functional phenotypic character, colour pattern variation, reflected the geographic patterns in the molecular data. Spatial-genetic patterns broadly match those in previously-studied, co-occurring low-mobility organisms, despite a variety of life histories. We suggest that for *E. rowelli*, the complex topography and history of the region has led to interplay among limited dispersal ability, historical responses to environmental change, local adaptation, and some resistance to free admixture at geographic secondary contact, leading to strong genetic structuring at fine spatial scale.

## Introduction

Morphologically cryptic diversity is important in conservation biology because failure to recognise the presence of distinct genetic groups can result in the loss of unique lineages not considered by management actions [1]. However, because it is not feasible to obtain information about genetic structuring in every taxon, a pragmatic approach is to identify, and base management on, the generalizable underlying organismal and environmental factors that generate biodiversity [2]. One powerful approach is to study multiple, broadly sympatric taxa, allowing the effects of organismal traits on spatial genetic structuring to be investigated in the absence of confounding environmental and community effects and different climatic histories [3].

**Figure 1:**
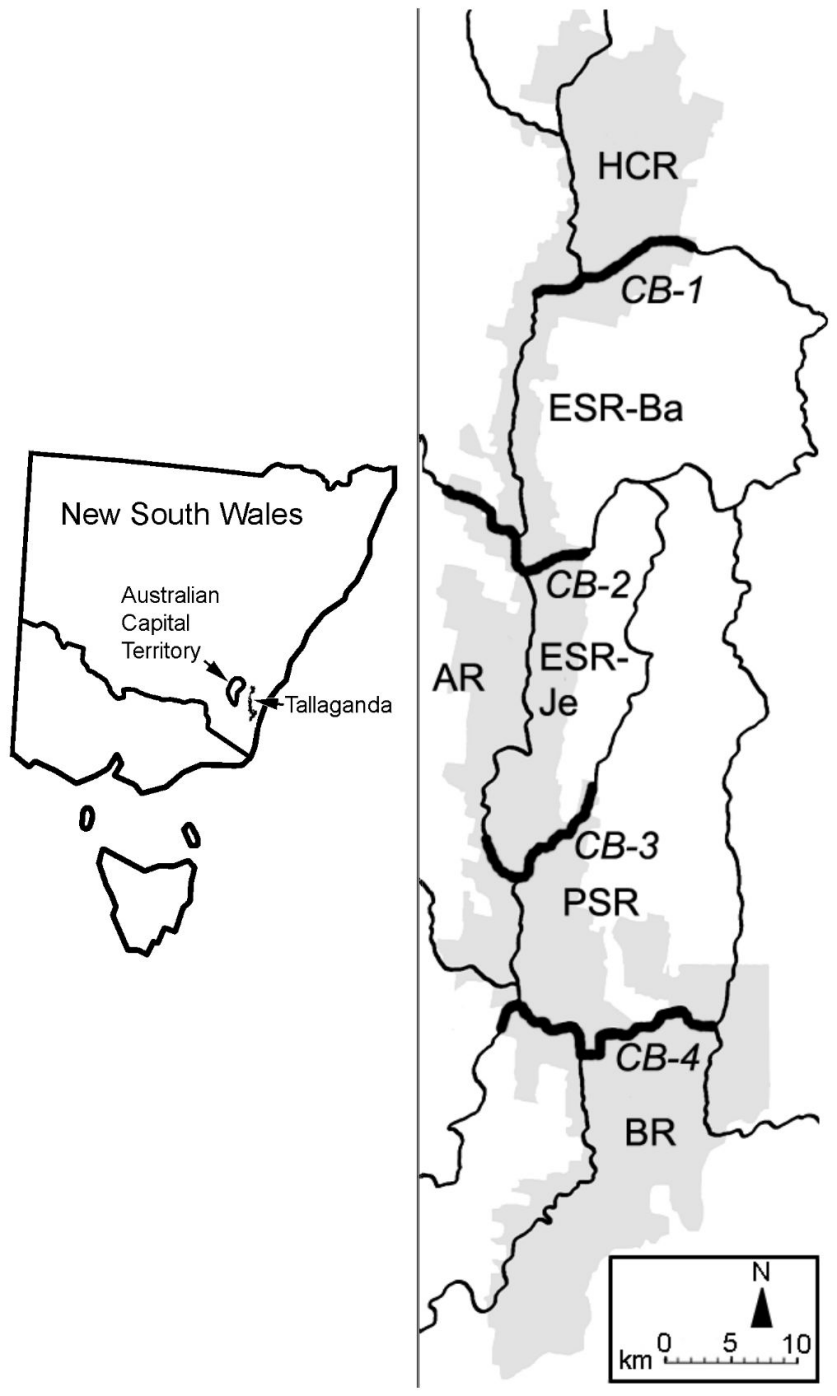
Location of the study region and layout of catchments. Location of Tallaganda in south-eastern Australia (left) and layout of catchments (right). Grey shading shows the extent of woody vegetation and black lines show ridges between catchments; those ridges used in testing the effects of catchments on genetic and phenotypic structuring are show as double thickness (labelled CB-1 to CB-4). Catchment labels (e.g. HCR) are acronyms; full names are given in the main text.

An appropriate landscape setting for investigating factors that generate biodiversity is the Tallaganda comparative phylogeographic study system described in Garrick et al. [4] and subsequent papers reviewed in Garrick et al. [5]. Briefly, Tallaganda is an area of mesic, eucalypt-dominated forest on a 100 km north-south section of the Gourock Range, a spur of the Great Dividing Range, in New South Wales, Australia (Figure 1). In addition to a north-south oriented Gourock Range ridgeline, a series of approximately east-west oriented ridges further subdivide the area into a series of microgeographic regions with distinct microclimates and hypothesized palaeoclimatic history [4,6,7].

The amplitude of late Pleistocene glacial cycling in the Southern hemisphere was less pronounced than that in the northern hemisphere [8]. Thus, rather than wide-ranging extinctions followed by recolonization from other areas, many Southern hemisphere species persisted within local refugia. Consequently, longer-term historical signals are likely to be retained in genetic data [8-10]. At Tallaganda, many saproxylic (i.e. species dwelling in decaying wood) and forest floor species are comprised of deeply-divergent genetic lineages, consistent with the persistence of woody vegetation in sheltered refugia, likely in deep gullies [5,11]. A notable and recurring pattern is evidence of phylogeographic regions that are broadly concordant across organisms encompassing a range of dispersal abilities and life histories [12,13]. These include Onychophora (velvet worms), including the species studied here [14,15], terrestrial Platyhelminths (flatworms) [13], Collembola (springtails) [4,12,16], funnel-web spiders [6,17], and skinks [7]. The phylogeographic regions identified correspond approximately but variably to five microgeographic regions proposed in Garrick et al. [4], which in turn are associated with topography and other environmental gradients that may have generated long-standing barriers to gene flow in saproxylic and forest-floor species [5,18]. These microgeographic regions have been named, north-to-south, Harolds Cross Region (HCR), Eastern Slopes Region (ESR), Anembo Region (AR), Pikes Saddle Region (PSR), and Badja Region (BR), and are referred to in-text by their acronyms (Figure 1).

The locally abundant *Euperipatoides rowelli* is a member of the phylum Onychophora, a group of soft-bodied terrestrial invertebrates accepted as the sister clade to the Arthropoda [19,20]. *Euperipatoides rowelli* is distributed over much of southern New South Wales, including Tallaganda, although morphological, chromosomal and genetic data indicate this range almost certainly includes multiple cryptic taxa [14,21,22]. Within Tallaganda, there is some evidence of life history differentiation among forms in different microgeographic regions [23], and morphologically aberrant individuals indicative of hybrid breakdown are found where at least one pair of regions meet [24]. Like most Australian Onychophora, *E. rowelli* is saproxylic and a predator of small invertebrates. Behavioural studies indicate they have complex social organization with a female dominated hierarchy [25]. Desiccation-resistance is limited due to spiracle-free trachea and the lack of a waxy cuticle, which together are unfavourable to dispersal owing to rapid water loss in dry conditions [26,27]. 

*Euperipatoides rowelli* is an important addition to the set of taxa studied at Tallaganda for at least two reasons. First, unlike the other invertebrate taxa studied in this system, there are detailed accounts of its life history, behaviour and dispersal [23,25,28,29]. Most recently, *E. rowelli* has being used as a model for evolutionary development, and will be the first Onychophora to have its full genome sequenced (http://www.arthropodgenomes.org/wiki/i5K) [30,31]. Second, it occurs at much higher densities than the other taxa studied in the system, sometimes exceeding 1000 individuals per hectare [26]. Since larger populations tend to lose diversity by drift more slowly than smaller ones, this species should retain genetic signatures of past climatic and geographic events for longer [1,5]. Moreover, the fact that this species is abundant over a large geographic range permits the collection of statistically informative samples with low risk of negative impacts on populations.

Onychophoran species diversity is likely to have been substantially underestimated [15,22,32,33], but the spatial-scale of such diversity remains unclear and may vary substantially depending on phylogeographic drivers. Most studies to date have focused on medium- to broad- spatial scales (but see 33,34,35), and have used DNA sequence markers, allozymes, karyotypes or combinations of these [21,32,35-41]. To complement these studies, we applied microsatellites to the fine-scale phylogeography of an Onychophoran that is extremely unusual in being sufficiently abundant to sample densely along a ~100 km transect. Microsatellite data are of particular interest because they capture variation on more recent time-scales than do markers used previously [42]. These data, together with mitochondrial (mt) DNA sequences and a phenotypic character (colour pattern variation) are used to gain insights into the responses of *E. rowelli* to environmental and paleoclimatic factors affecting forest distributions at Tallaganda throughout the Pleistocene and earlier. We test the hypotheses that (1) major genetic groups will be present within *E. rowelli* at the catchment-scale (i.e. associated with watersheds) and (2) they will broadly correspond to microgeographic regions of the Garrick et al. [4] model.

## Methods

### Sampling

*Euperipatoides rowelli* individuals were collected by hand from decaying logs throughout Tallaganda between 1989 and 2009, with overlapping sets of individuals used for microsatellite, mtDNA and colour pattern analyses. Samples were collected on public land, under permits issued to NN Tait and named associates, renewed annually, covering all years of collection, by Forest New South Wales State Forests (XX51212 covering Southern and Monaro Regions) and New South Wales National Parks (SL100159 covering Tallaganda). For each log, latitude and longitude were recorded, and it was classified into one of the microgeographic regions of Garrick et al. [4]. Sampling locations for each dataset are given in Table S1.

Individuals from single logs were collected into containers with rotting wood to maintain humidity. Subsequently, individuals were weighed to the nearest milligram, and sex was determined according to presence or absence of male-specific crural papillae: individuals >25 mg without crural papillae were classified females based on dissection data [23], and individuals <25 mg lacking crural papillae were classified as of unknown sex. Some individuals were scored for phenotype as described below then all individuals were frozen at -80 °C.

### DNA Extraction and Amplification

DNA was extracted from ~40 mg tissue per individual using a salting-out [28] or modified CTAB [43] protocol; the PCR-inhibitor-containing skin was removed prior to extraction. Eight microsatellite loci, including six new loci developed from 454 pyrosequencing data (Dryad Digital Repository doi:10.5061/dryad.f1cb2/43) [44,45] using Primer v3 [46], were scored for 636 individuals from 73 sites (mean ± standard deviation = 8.7 ± 3.1 individuals per site, range = 1–19, this notation is used for summary statistics in brackets below; sample sizes per site given in Table S1 for all data types). Sites were defined as 1 to 3 logs with < 100 m between the furthest apart, and were separated from other sites by distances > 100 m, although typically inter-site distances were much larger; multi-log sites were used to allow the inclusion of logs with low sample sizes. Microsatellites were amplified as two multiplexes, each containing 1x Multiplex PCR Master Mix (QIAGEN), between 0.025 and 0.1 μM of forward and reverse primers for each locus, 0.03 μM of each of two fluorescent-dye-labelled M13 primers [47], and ~350 ng of genomic DNA. Thermocycling conditions for both multiplexes were 95 °C for 15 min, 33 cycles of 94 °C for 20 s, 90 s annealing, and 71 °C for 60 s, followed by 60 °C for 30 min. Annealing temperature was 63 °C for the first cycle and decreased by 2 °C per cycle for the next 4 cycles, with the remaining cycles at 55 °C. Primer sequences, sources, and multiplex details are given in Table 1. Microsatellites were scored using a LI-COR Global IR2 two-dye DNA Sequencer (model 4300) with electrophoresis on 6% polyacrylamide gels.

**Table 1:** Microsatellite loci used in this study, with details of their use in multiplex PCR reactions.

**Locus**	**Primer sequences (5' -> 3') *^1^***	**Repeat**	**Multiplex**	**Dye**	**[uM]**	**# alleles**	**bp Range**	**Global F_ST_**	**GenBank**
19 **^*2*^**	AACCTAACCGCTCCTCCCTA	AC	A	700	0.05	7	239-251	0.59	KF373246
	TCTGTAGGGTCCTCTCCACCT								
29 **^*2*^**	CTGGTGTGAACGCTATAGACC	AG	B	700	0.025	8	181-197	0.43	KF373247
	AACACACTTTCTCATCTAGAGTCATC								
39 **^*2*^**	TCCTTAATTGGGCGAGAAAGC	AAT	B	700	0.1	13	217-250	0.44	KF373248
	TTCCGGTTCCTGTTTTGGC								
57 **^*2*^**	TGCGTAATGACTGCATGGTTG	ATT	B	800	0.025	10	242-269	0.44	KF373249
	AGTGCCTTGAGGAAGGTCAG								
62 **^*2*^**	TCATGCCCTTATGTCCAGAG	GAT	A	700	0.05	4	210-219	0.59	KF373250
	GACAAATAACGAAGTCTTTACACG								
64 **^*2*^**	TAGGCGATTCTCATGGCTTG	ATT	B	800	0.1	11	187-221	0.29	KF373251
	TTCCATATCAGTTAGGGCTAGGA								
p06new **^*3*^**	CACACACACTAGTTTGCCTAAG	AC	A	800	0.1	4	211-225	0.59	AF109350.1
	CAAACATGTATGGTATTTG								
p17low **^*3*^**	TGTTTCCATCCAGGTTCCAAT	AC	A	800	0.1	3	118-123	0.58	AF109351.1
	TGTATCAAAAGACATGGACAGG								

^1^ M13 tail concatenated to 5’ end of each forward primer for use in visualising not shown.

^2^ New locus designed during this study from 454 pyrosequencing data

^3^ Redesigned versions of primers for loci described in Sunnucks & Wilson [14] which give improved performance.

Sequence variation in a 448 bp portion of the mitochondrial cytochrome c oxidase (COI) gene was assayed for 601 individuals from 57 sites (10.5 ± 13.5, 1 - 96) using a combination of Single Stranded Conformational Polymorphism (SSCP) assays [48], and direct sequencing performed by Macrogen, Korea. Amplification used primers C1-J-1718 and C1-N-2191 [49] following [13]. Cycling conditions were 2 min at 94 °C, 35 cycles of 94 °C for 20 s, 50 °C for 30 s and 72 °C for 45 s, followed by 2 min at 72 °C. Sequences were edited and aligned using MEGA v4.0 [50]. All sequences could be translated into open-reading frames, and patterns of variation were consistent with functional mtDNA (Results), indicating that mtDNA was amplified [51]. Unique haplotypes have been deposited in GenBank (Accession numbers: JQ582751 - JQ582768, KC810894 – KC810942).

### Scoring of body colour pattern

Common-garden culture experiments indicate that body colouration in *E. rowelli* is under genetic control (P. Sunnucks, unpublished data). Three colour pattern traits were scored for 1030 adults from 92 sites (11.3 ± 13.6, 1–89) and their geographic distributions subsequently compared with genetic data and the microgeographic-region model for Tallaganda [4]. Traits were colour of segmental spots (orange - state 1, white - 2, absent - 3), colour of additional spots (orange - 1, white - 2, absent - 3) and pattern of additional spots (two distinct rows - 1, two distinct rows with small spots scattered along the dorsal midline - 2, two distinct rows with small spots in a diamond pattern along the dorsal midline - 3, absent - 4). Throughout the text, the combined phenotype of individuals is recorded as a 3 digit number comprising the state-codes at each trait, in the order above. Juveniles were not scored because they display distinctive phenotypes that differ from their final phenotype (P. Sunnucks, unpublished data).

Statistical analyses were performed to determine the presence of geographic structure in the microsatellite, mtDNA and colour pattern data separately, and to determine if these patterns were concordant among the three data types, and with the *a priori* microgeographic-region model.

### Statistical analyses - patterns in single variables

To ensure microsatellite data were appropriate for analyses, linkage disequilibrium and departures from Hardy-Weinberg equilibrium were assessed using GENEPOP v4.0 [52] with sites as population units. To determine the number of nuclear genetic groups sampled, microsatellite data were analysed using STRUCTURE v 2.3.3 [53]. The potential number of genetic groups (K) examined were 1 to 10, with burn-in and run lengths of 1x10^6^ and 3x10^6^ MCMC generations respectively per run, with 20 replicate runs for each K-value. The admixture model and correlated allele frequencies were used with other settings left as default; location priors were not used. The ΔK method [54] was used to select the best-fit number of genetic groups. Individuals were assigned to a group if they had membership coefficients (Q values) to it > 0.8 in the mostly likely run (i.e. that with the lowest Ln PrPr(X | K)); individuals with Q < 0.8 were unassigned. Where multiple runs were equally likely at the chosen K, one was chosen at random. For consistency with previous studies at Tallaganda, inferred K-value was also examined following Pritchard et al. [53].

As the ΔK method detects the highest level of structure in hierarchically structured populations [54], each group identified was reanalysed using STRUCTURE, as above, testing K 1 to 6. This was repeated until no further substructure was identified. Similar approaches have previously been used where hierarchical genetic structure is likely [54,55,56]. STRUCTURE may produce spurious clustering under some circumstances, such as under isolation-by-distance (IBD) with patchy sampling [58]. Mantel tests for IBD were performed in GENEPOP to examine whether groups may have arisen spuriously. To determine whether among-group differences had arisen over long-term evolutionary timescales where mutational changes accumulate, versus short-term ecological timescales where drift is the predominant force, pairwise pR_ST_ - R_ST_ tests between groups were performed in SPAGeDi v1.3 [57]. 

To estimate relationships among mtDNA haplotypes, a phylogenetic tree was inferred using MrBayes [58] under the HKY+I+G model of molecular evolution. This was chosen in preference to the GTR model, whose assumption that all transitions and transversions occur at appreciable rates [58] was violated (data not shown). Different codon positions were allowed to evolve at different rates to account for different degeneracies [59]. Runs were performed assuming clock-like and non-clock-like evolution: both gave very similar results (data not shown), so we report only the non-clock-like results. The following outgroups were included: *Euperipatoides leuckartii* (GenBank accessions: EU855390.1 and U62426.1), *Ruhbergia bifalcata* (AF337996.1) and *Ooperipatus* sp. (AF337994.1). Where nuclear microsatellite data were available for *E. rowelli* individuals with mtDNA sequence data, STRUCTURE clusters were overlaid on the phylogenetic tree, as was the presence of each haplotype in five major geographic regions delimited by common mtDNA haplotypes, which corresponded roughly to the *a prior* regions (Results). Tajima’s D [60], Fu’s Fs [61], ratio of synonymous to non-synonymous mutations, and pattern of mutations across codon positions were calculated across all observed haplotypes using DnaSP v5.1 [62]. Aligned sequences were translated to amino acids in GENEIOUS v6.1.6 together with all other available onychophoran COI sequences (n = 421 as of 21 October 2013) to compare patterns of amino acid variation with other taxa in the phylum, and with a model of insect COI evolution [63].

To determine how the three colour-traits combined into multi-trait phenotypes, a log-linear model [64] was used to test the independence of the three traits across all individuals scored. This and subsequent tests were performed in R [65], with α = 0.05 significance level.

### Statistical analyses – correspondence of patterns across variables

Two approaches were used to determine correspondence of patterns among data types and to microgeographic region boundaries. First, Fisher’s exact tests [66] for two-way contingency tables were used to test if mtDNA haplotype and nuclear assignment group were associated among individuals. This was repeated for colour pattern phenotype and STRUCTURE group based on nuclear microsatellites; colour pattern phenotype was analysed as a multi-trait phenotype. As associations across data types largely reflected the hierarchical relationships among nuclear genetic groups, tests were repeated at each level of the hierarchy where the overall test was significant; Bonferroni corrections [64] were used to correct for multiple tests. Association between colour pattern phenotype and mtDNA haplotype, and among all three variables, was not assessed as few individuals had all data. Only individuals with mtDNA haplotypes seen in > 9 individuals (> 76 % of all individuals - ‘common’ haplotypes hereafter, all other haplotypes ‘rare’), phenotypes seen in > 9 individuals (> 97 %) and unambiguous nuclear assignment groups (> 61 %) were considered in this analysis and in the analysis following.

Second, boundary overlap analysis [67] was used to test for non-random association of genetic and/or colour pattern phenotype spatial discontinuities with a key physiogeographic landscape feature – high-elevation ridgelines. To reduce the complexity of these analyses, we focused on four major east-west oriented major catchment boundaries tested in previous work (CB-1 to CB-4, Garrick et al. [12]). For response variables, we considered coarse genetic breaks characterized in three ways (1. zones of rapid change in STRUCTURE clusters as defined by the second hierarchical level, 2. major mtDNA clades, or 3. common mtDNA haplotypes) and breaks in colour pattern phenotypes. Given that different contact zone dynamics may be operating across the range of a species [68], two separate sets of analyses were performed. First, a separate overlap analysis per single catchment boundary was performed on each dataset. Second, we reanalyzed these data considering all four boundaries simultaneously to understand if catchment boundaries have pervasive impact on one or more data types at the level of the whole forest.

Boundary overlap analysis involves two steps: identification of geographic zones of rapid change in the value of one or multiple variables relative to neighbouring sites, and evaluation of whether boundaries coincide to a significant extent [69,70]. *Step 1: Boundary detection*. We used categorical wombling [71]: each site was assigned to its associated catchment, with sites within 100 m of ridgelines randomly assigned to one of the most proximate catchments [13]. Using a majority-rule approach, each site was also assigned to a single STRUCTURE cluster, mtDNA clade, common mtDNA haplotype, and colour pattern phenotype. Next, adjacent sites were connected using Delaunay triangulation. For each linked pair of sites, a Boundary Likelihood Value (BLV) was calculated for the relevant categorical variable (i.e. population or catchment membership). Geographic locations with highest rates of change were identified as Boundary Elements (BEs; Voronoi edges drawn perpendicular to connections in the Delaunay network, equidistant from the two nearest sites). Threshold BE values were set at natural breaks in the BLV distribution. Finally, adjacent BEs were linked to form spatially contiguous population or catchment boundaries. *Step 2: Testing for directional overlap*. The statistic *O*_*1*_ (mean geographic distance of BEs in Boundary 1 to the nearest BE in Boundary 2) was used to test the hypothesis of no association between / among boundaries. To generate null distributions of *O*_*1*_, 10,000 Monte Carlo randomizations were performed under complete spatial randomness permutation in BOUNDARYSEER v 1.3.14 (Biomedware). Because catchment boundaries are independent variables that may influence the location of contact zones, only the latter were randomized. We assessed significance of observed test-statistics relative to the lower tail at the 0.05 level.

## Results

### Nuclear genetic structure

 All individuals were successfully genotyped for all loci. Between three and 13 alleles were detected at microsatellite loci (mean = 7.5; Table 1). Linkage disequilibrium was significant (uncorrected P < 0.05) in only 27 (2.5 %) of 1088 locus-pair-site combinations. Thirteen significant values came from 3 sites later identified to have many putative hybrids, and there was no clear pattern of certain sites or loci being repeatedly involved in remaining significant tests. Significant departures (uncorrected P < 0.05) from Hardy-Weinberg equilibrium, all but one heterozygote deficits, were detected in 63 (10.8 %) of 584 locus-site combinations, with all loci showing significant deviation from expected genotype proportions in at least one site. Wahlund effects (the presence of multiple genetic groups within a sampling location) is a more likely cause than are null alleles, because all but four significant departures occurred at sites identified to have individuals from multiple genetic groups or putative hybrids (below). 

**Figure 2:**
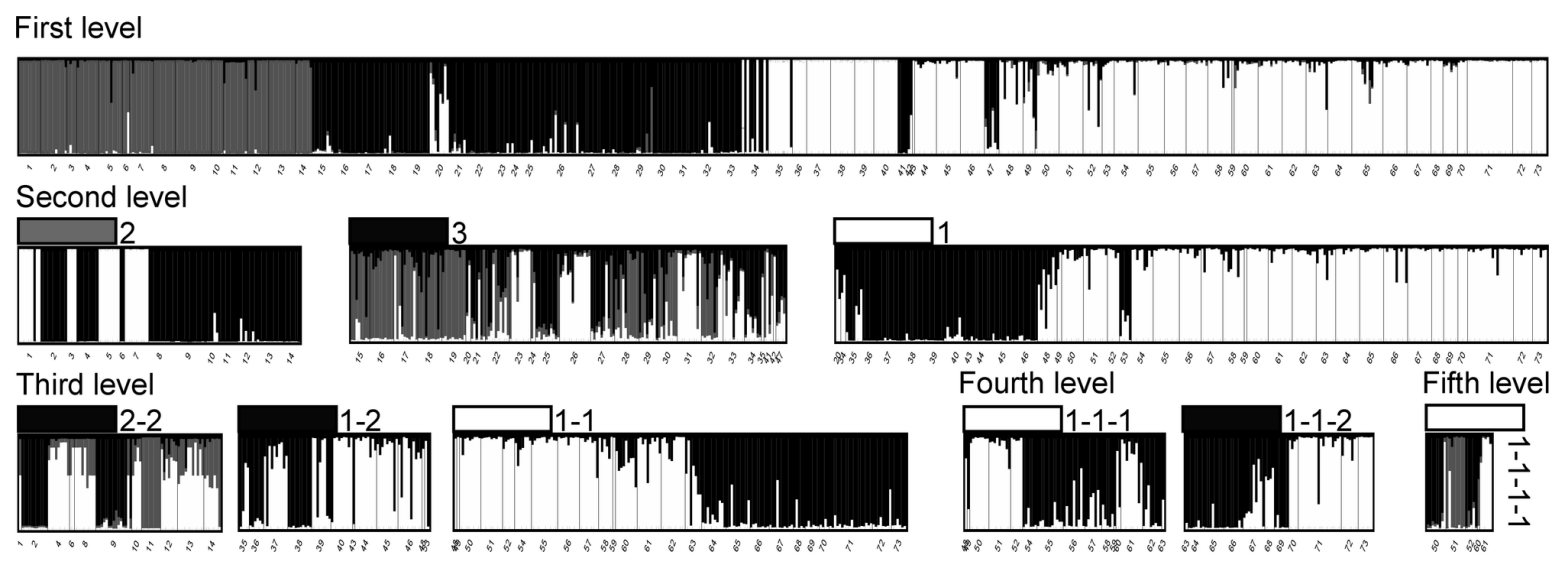
Patterns of assignment to nuclear genetic groups in *Euperipatoides rowelli* individuals within Tallaganda. Assignment probabilities for individuals at each level in a hierarchical STRUCTURE analysis. Level 1 refers to an analysis including all individuals, and subsequent levels involve runs with individuals assigned to a single group (Q > 0.8) in the level above. Colour patches indicate which group in the previous level each STRUCTURE plot shows, and numbers show the hierarchical relationships among groups (e.g. groups 1-1 and 1-2 are nested within group 1). Numbers under the plots identify sites individuals came from (given in Table S1).

**Figure 3:**
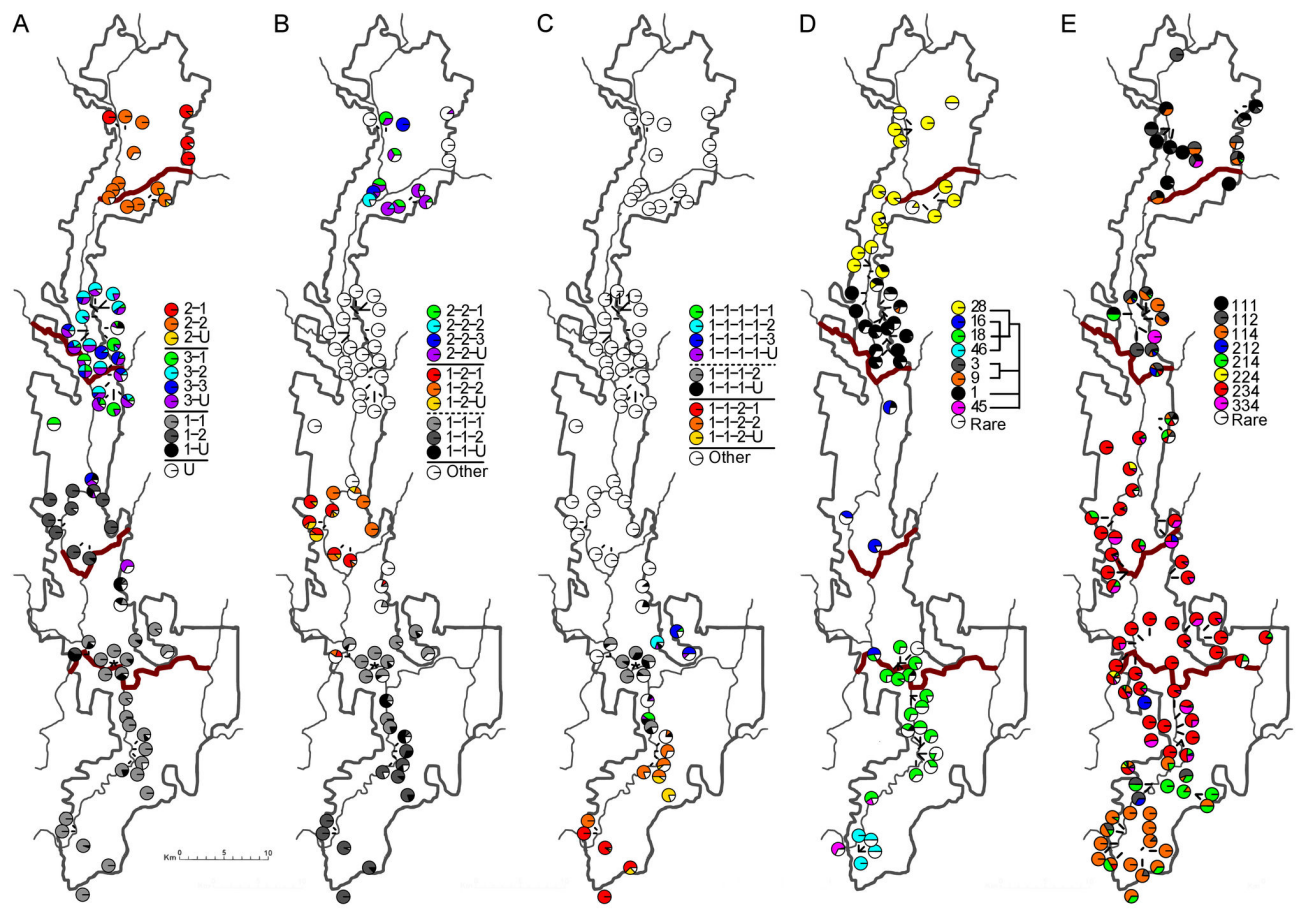
Geographic distribution of genetic and phenotypic traits for *Euperipatoides rowelli* individuals within Tallaganda. (A, B, C) Nuclear genetic groups identified by a hierarchical STRUCTURE run; numbers show the hierarchical relationships among groups (e.g. – groups 1-1 and 1-2 are both nested within group 1) with ‘U’ representing unassigned (Q < 0.8 for all identified clusters) at that level of the hierarchy. Solid and dotted bars in the legends represent differences at different levels of the hierarchy. (D) Common mtDNA haplotypes; phylogeny on the legend shows the relationships between these and is simplified from a formal Bayesian analysis of all observed haplotypes (Figure 4). (E) Common phenotypes; described by three non-independent colouration traits defined in the text. Each pie chart represents a site of 1 - 96 individuals; some sites consist of multiple logs, with no more than 100 m between the furthermost. Details of sample numbers given in Table S1. The thick dark-red lines show catchment boundaries used in some analyses. Catchment boundaries are not shown on subfigures B and C as these data were not analysed with regards to these boundaries.

STRUCTURE analysis identified 15 nuclear genetic groups, with up to five levels of nesting (Figure 2). Strong structure is reflected in the fact that only 42 individuals (6.6 %) were unassigned at the first level of nesting. At all levels, members of each genetic group were spatially aggregated and unassigned individuals were typically sampled from spatial locations at the edges of groups or where groups met, consistent with genuinely admixed individuals (Figure 3A-C). The geographic distribution of the three major nuclear genetic groups (those at the first hierarchical level) approximated the regions HCR (group 2), ESR (group 3) and combined PSR-AR-BR (group 1). In this last group, AR (group 1-2) is less genetically similar to PSR and BR (group 1-1) than the latter are to each other. Areas of transitions between groups usually corresponded to microgeographic region boundaries but some groups crossed boundaries (group 2 in HCR and ESR-Ba, suffixes refer to subcatchments, Figure 1), and conversely some transitions occurred in the absence of catchment boundaries (group 2 to group 3 transition within ESR-Ba, group 3 to 1 transition within ESR-Je). Two groups clearly deviated from the general pattern of genetic groups fitting catchments: there were spatially unstructured sub-groups within groups 3 and 2-2. Little or no IBD was detected in these two groups (P = 0.07 and P = 0.57 respectively), indicating they are not merely artefacts of sampling along a genetic gradient.

Despite the strong spatial nuclear-genetic differentiation, STRUCTURE analysis identified six individuals with highest cluster membership to a geographically distant cluster compared to the one typical of their sampling location. First, a male and an individual of unknown sex assigned to group 1-U were sampled among group 3 individuals 16 km north of the closest other group 1 individual. Second, a male and three females assigned to group 3-U were found among group 1 individuals 10 km south of the closest sampled group 3 individual (Figure 3A-C). All alleles in the individuals with locally unusual genotypes were seen in other local individuals, calling for some circumspection in inferring long-distance dispersal.

There were qualitative differences in apparent timing of genetic divergence at the different hierarchical levels, as indicted by pR_ST_ – R_ST_ tests. At the top level of the nuclear-hierarchy, pR_ST_ – R_ST_ tests indicated that two pairs of groups (1 and 2, 1 and 3) diverged over timescales sufficient for microsatellite evolution (both P = 0.02). Conversely, divergence between groups 2 and 3 was explicable by recent genetic drift (P = 0.42). Nearly all pairwise pR_ST_ – R_ST_ tests were non-significant at lower hierarchical levels, with none significant after correcting for multiple tests, also indicating a drift timescale (Table 2).

**Table 2:** Uncorrected P-values for pairwise pR_ST_ – R_ST_ tests between nuclear genetic groups identified in *Euperipatoides rowelli* individuals within Tallaganda by a hierarchical STRUCTURE method. The test is permutation based and has no associated test statistic. Bold values significant at α = 0.05.

	**1-1-1-1-1**	**1-1-1-1-2**	**1-1-1-1-3**	**1-1-1-2**	**1-1-2-1**	**1-1-2-2**	**1-2-1**	**1-2-2**	**2-1**	**2-2-1**	**2-2-2**	**2-2-3**	**3-1**	**3-2**	**3-3**
**1-1-1-1-1**	0.00														
**1-1-1-1-2**	0.43	0.00													
**1-1-1-1-3**	0.79	0.62	0.00												
**1-1-1-2**	0.39	0.24	0.38	0.00											
**1-1-2-1**	**0.03**	0.20	0.53	0.19	0.00										
**1-1-2-2**	**0.04**	0.20	0.20	0.17	0.18	0.00									
**1-2-1**	0.60	0.69	0.92	0.48	0.66	0.29	0.00								
**1-2-2**	0.45	0.41	0.57	0.24	0.39	0.08	0.75	0.00							
**2-1**	0.16	0.29	0.32	0.13	**0.02**	0.08	0.18	0.11	0.00						
**2-2-1**	0.07	0.40	0.32	0.11	**0.02**	0.17	0.18	0.09	0.77	0.00					
**2-2-2**	**0.01**	0.31	0.38	0.14	0.07	0.22	0.29	0.14	0.65	0.34	0.00				
**2-2-3**	0.12	0.54	0.41	0.06	**0.01**	0.25	0.29	0.10	0.94	0.73	0.71	0.00			
**3-1**	**0.03**	0.36	0.18	0.06	**0.03**	0.52	0.26	0.06	0.52	0.13	0.20	0.59	0.00		
**3-2**	**0.00**	0.15	**0.02**	0.05	**0.02**	**0.05**	0.10	**0.05**	0.16	0.18	0.51	0.62	0.05	0.00	
**3-3**	0.07	0.17	**0.02**	**0.05**	0.05	0.30	0.12	**0.04**	0.52	0.37	0.71	0.86	0.45	0.77	0.00

Four genetic groups were indicated by the method of Pritchard et al. [53], corresponding to HCR (group 2 in the previous analysis), ESR (group 3), AR (group 1-2) and a combined PSR-BR group (group 1-1) (data not shown); an outcome consistent with results of the hierarchical approach.

### Mitochondrial COI

MtDNA diversity was substantial; the 601 individuals yielded 66 unique haplotypes, with 154 variable sites of which 116 were parsimony-informative. Synonymous differences were far more common than non-synonymous (172 to 36), with 26 first-position, 15 second-position, and 113 third-position sites variable. Tajima’s D was not significantly different from zero when analysed across all sequences (D = 0.87, P > 0.10), nor was Fu’s Fs (Fs = -0.92, P > 0.10), providing no evidence of strong nucleotide selection or population growth. Haplotype frequencies ranged from 0.17 % to 26.47 %, with eight being common (observed in > 9 individuals), and differed by 0.22 % to 15.40 % uncorrected *p*-distance. Overall, 32 of 149 amino acid sites in the alignment were variable. Most (25) of these occurred in very few individuals, but seven among common haplotypes. These common *E. rowelli* protein variants may not be strongly constrained by function: six residues (amino acids #108, 111, 112, 135, 174 and 175) also vary among insects [63], and the seventh (107) is variable among the Onychophora listed in Methods. In contrast, the set of five common haplotypes diagnostic of each microgeographic region (below) encoded the same protein sequence despite DNA sequence differences of up to 6.47 %, suggestive of selection maintaining high frequencies of a particular protein sequence [63].

**Figure 4:**
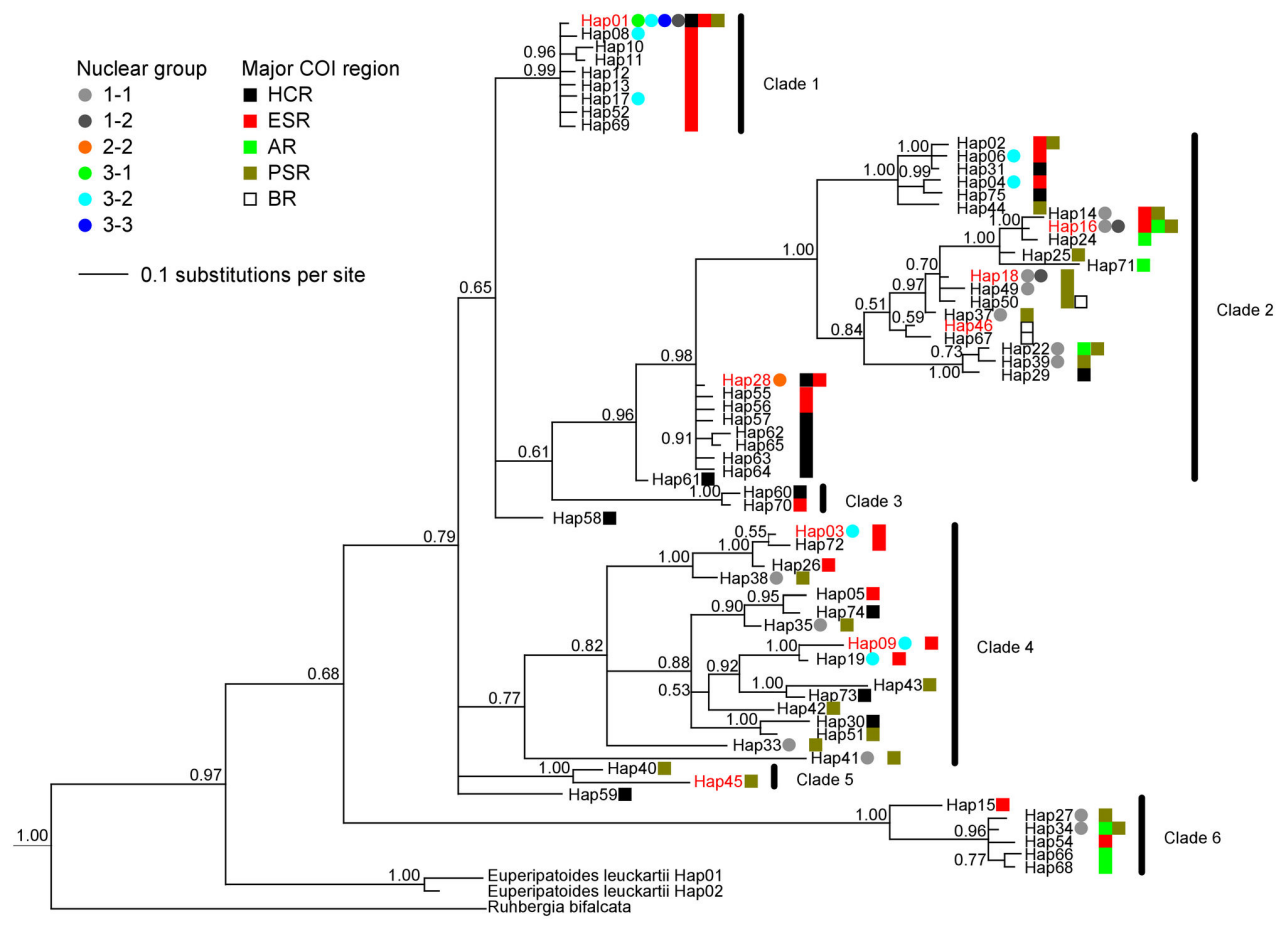
Phylogeny of partial COI mtDNA haplotypes in *Euperipatoides rowelli*. A non-clock HKY+I+G model of evolution was used. Numbers at the root of each branch are Bayesian posterior probability support values. Haplotype labels in red are those which are common (>9 occurrences across all individuals). Dots next to haplotype labels represent second-level nuclear assignment group of individuals with those haplotypes. Squares next to haplotype labels represent occurrence of that haplotype in each of five regions defined on the spatial distribution of the common haplotypes, which correspond roughly to the five microgeographic regions of Garrick et al. [4]. Clades defined by the first branches in from the outgroups with support values > 0.75. Haplotypes 58 and 59 are not members of any clade. Phylogeny is rooted with an additional outgroup (*Ooperipatus* sp.) not shown here.

The inferred mtDNA phylogeny had strong structure: six distinct clades had Bayesian posterior probability values > 0.75, including a basal clade on a very long branch (Figure 4). For each clade where nuclear genetic assignments of some individuals was available, multiple nuclear genetic groups were represented within clades but each haplotype was found associated with only a single nuclear group at the first level of the hierarchical STRUCTURE analysis. Common haplotypes were distributed throughout the phylogeny. 

Generally, mDNA haplotypes were strongly spatially structured: five major regions corresponding broadly to *a priori* microgeographic regions each had a single very common haplotype along with up to three other common haplotypes (Figure 3D). As with nuclear groupings, regional contact zones had some sites containing haplotypes from both regions. 

The spatial distribution of the eight common mtDNA haplotypes reflected their phylogenetic relationships, except that northernmost haplotype 28 (HCR) was more closely related to southernmost sequences (AR-PSR-BR) than to neighbouring common haplotype 1 (ESR) (Figure 3D). Rare haplotypes showed little spatial structuring and often occurred in different regions than their most similar common haplotype (Figure 4). However, over 70 % of rare haplotypes were detected in only one or two individuals, so their distributions are uncertain. Where common haplotypes were found in multiple regions, it was always adjoining ones; this was also true at the level of the clades, which were usually spatially centred on the region bearing their common haplotype.

### Colour pattern variation

The three colour patterns traits were distributed as a few discrete phenotypes rather than varying independently (G^2^_28_ = 0.94, P = 0.33 for three-way interaction, G^2^_>29_ > 239.17, P < 0.001 for all two-way interactions). Nearly all (> 97 %) individuals had one of eight common phenotypes, with the remainder being spread over 11 rare phenotypes. Colour pattern phenotype was much less spatially structured than were nuclear groups or mtDNA haplotypes: a given area was less strongly characterised by a single phenotype and the transitions between areas, especially in the northern half of the study area, were less sharp than for genetic markers (Figure 3E). Broadly, HCR and ESR were characterised by phenotypes with orange segmental and body spots, with HCR having a higher frequency of individuals with patterned body spots. AR and PSR were similar to each other, characterised by white or absent segmental spots and no additional spots. BR was almost exclusively occupied by individuals with orange segmental and body spots but no additional pattern, similar to some ESR individuals. The group of ‘214’ colour pattern individuals between PSR and BR can be interpreted as hybrid phenotypes, with the white segmental spots of PSR, and orange body spots but no additional patterned spots of BR. The greater similarity in phenotypes between AR and PSR than PSR and BR is inconsistent with the relationships indicated by STRUCTURE analysis, which suggests a closer grouping of PSR and BR (above).

### Correspondence of variables

**Figure 5:**
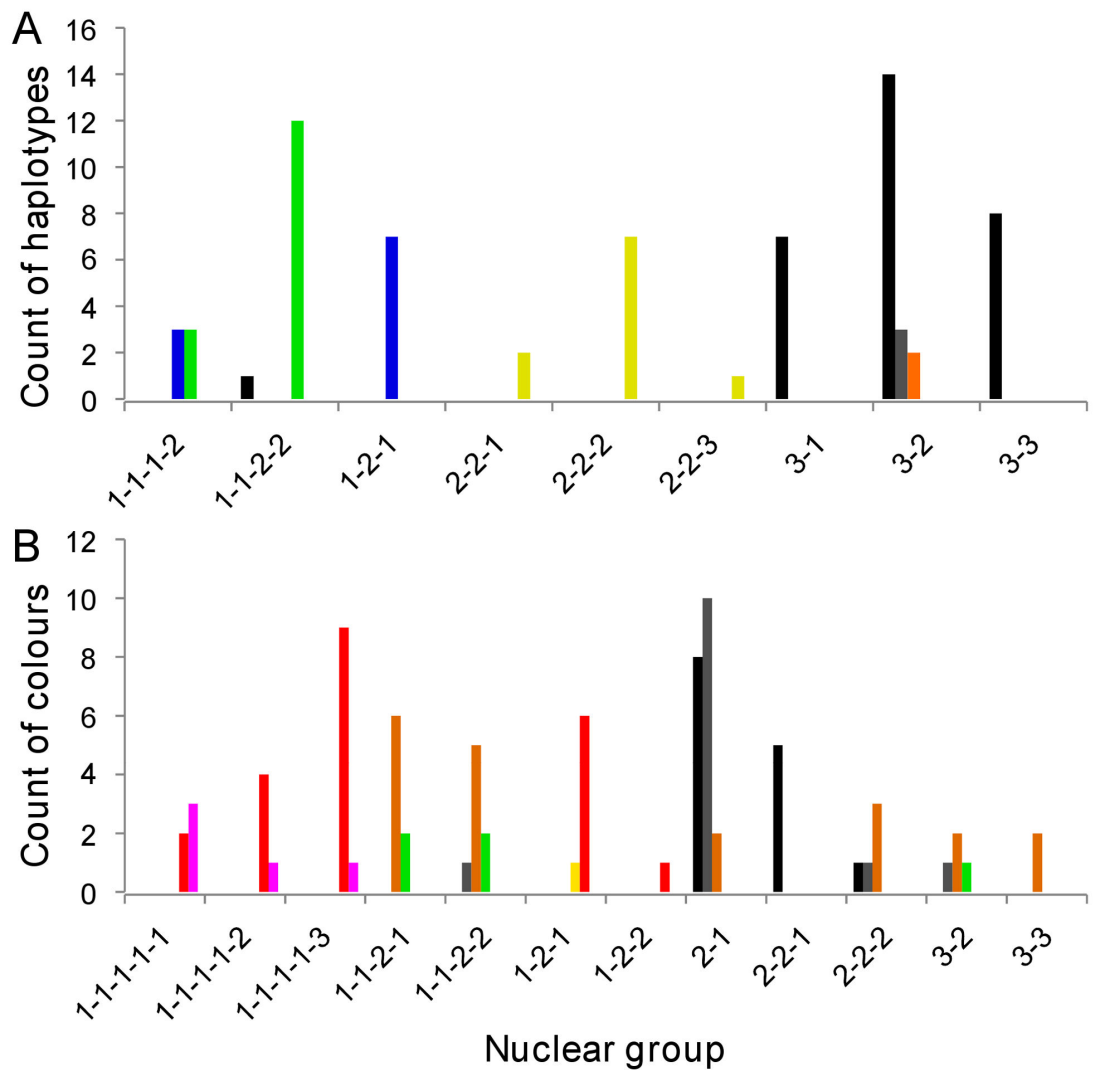
Association between nuclear genetic, mitochondrial genetic and colour pattern phenotype in *Euperipatoides rowelli*. Distribution of (A) common mtDNA haplotypes and (B) common phenotypes across nuclear assignment groups; numbers show the hierarchical relationships among groups (e.g. groups 1-1 and 1-2 are both nested within group 1). Legends to haplotypes / colour pattern phenotypes follows Figure 3.

There was a significant association overall between mtDNA haplotype and nuclear group, and between colour pattern phenotype and nuclear group (both P < 0.001) (Table 3, Figure 5). When examined with respect to the hierarchical nature of the nuclear groups, these associations held at the uppermost level and for one or two levels within nuclear group 1, but not otherwise (Table 3).

**Table 3:** Corrected P-values for Fisher’s exact tests for positive associations between (a) common mtDNA haplotypes and (b) common phenotypes across nuclear assignment groups, at different levels within the identified hierarchy.

**(a) Mitochondrial – Nuclear**			**(b) Colouration - Nuclear**	
**Analysis**	**Corrected P-value**		**Analysis**	**Corrected P-value**
1 v 2 v 3	**< 0.001**		1 v 2 v 3	**< 0.001**
1 v 2	**< 0.001**		1 v 2	**< 0.001**
1 v 3	**< 0.001**		1 v 3	**0.012**
2 v 3	**< 0.001**		2 v 3	0.088
1-1 v 1-2	**< 0.001**		1-1 v 1-2	**0.039**
1-1-1 v 1-1-2	0.138		1-1-1 v 1-1-2	**< 0.001**
1-2-1 v 1-2-2	Unable to test		1-1-1-1 v 1-1-1-2	Unable to test
2-1 v 2-2	Unable to test		1-1-2-1 v 1-1-2-2	1.000
2-2-1 v 2-2-2 v 2-2-3	1.000		1-2-1 v 1-2-2	1.000
3-1 v 3-2 v 3-3	0.500		2-1 v 2-2	0.061
			3-2 v 3-3	1.000

It Was Not Possible to Perform Some Tests Due to the Lack of Individuals with Haplotype / Phenotype Data within Some Nuclear Assignment Groups. The Test Is Permutation Based and Has No Associated Test Statistic. P-Values Have Been Bonferroni Corrected for the Number of Tests at That Hierarchical Level. Bold Values Significant at α = 0.05

### Spatial correspondence of contact zone boundaries versus catchment ridgelines

Catchment boundary 2 (CB-2), which runs east-west along the Ballalaba (ESR-Ba) and Jerrabatgulla (ESR-Je) watershed and bisects ESR, was the strongest predictor of contact zone boundaries seen in *E. rowelli*. Major mtDNA clades (reflecting divergences occurring over evolutionary timescales), common mtDNA haplotypes (reflecting shorter-term processes of population differentiation dominated by genetic drift), and colour pattern phenotype-based contact zones all showed significant directional overlap with CB-2 (*P* < 0.05, Table 4). The contact zones between nuclear genetic groups (second hierarchical level, Figure 3A) were not significantly associated with CB-2. A significant break in nuclear genotypes did coincide with CB-3 (the division between ESR and PSR, i.e. the transition from cluster 1-2 to 1-1; *P* < 0.001, Table 4). All other tests of directional overlap indicated non-significant spatial associations between *E. rowelli* contact zones and catchment boundaries.

**Table 4:** Boundary overlap analysis of *E. rowelli* contact zones and hypothesized topographic barriers (CB-1 to CB-4).

	Directional overlap	Boundary set		*P*-value
		Boundary 1 (response)	Boundary 2 (predictor)	
	STRUCTURE 2^nd^	cluster 2 / cluster 3	CB-1	0.064
	level clusters	cluster 3 / cluster 1-2	CB-2	0.999
	vs. catchments	cluster 1-2 / cluster 1-1	CB-3	***< 0.001***
		cluster 1-2 / cluster 1-1	CB-4	0.968
		clusters-all	CB-all	0.707
	MtDNA major clades	clade 2^North^ / clade 1	CB-1	0.983
	vs. catchments	clade 1 / clade 2^Central^	CB-2	***< 0.001***
		clade 1 / clade 2^Central^	CB-3	0.991
		clade 1 / clade 2^Central^	CB-4	0.962
		clades-all	CB-all	1.000
	MtDNA haplotypes	hap. 28 / hap. 1	CB-1	0.963
	vs. catchments	hap. 1 / hap. 16	CB-2	***0.001***
		hap. 16 / hap. 18	CB-3	0.147
		hap. 18 / hap. 46	CB-4	0.978
		haplotypes-all	CB-all	0.412
	Colour pattern	111+112+114 / 111+112+114	CB-1	0.857
	phenotypes	111+112+114 / 234	CB-2	***0.036***
	vs. catchments	234+334 / 234	CB-3	0.097
		234 / 112+114+214+234	CB-4	1.000
		phenotypes-all	CB-all	0.984

Nuclear genotypic groups and mtDNA clade names follow Figures 2 and 4, respectively, and “/” represents the associated contact zone. Joint analysis of overlap among all contact zones and catchment boundaries for a given data type are indicated by the prefix “-all”. *P*-value represents significance of the test statistic at the lower 0.05 level.

## Discussion

Strong, locally endemic genetic structure was seen for *Euperipatoides rowelli* at Tallaganda. Nuclear, mitochondrial and phenotypic data identified distinct lineages distributed generally within catchments or subcatchments despite continuous habitat.

These patterns of biodiversity are markedly different to those seen elsewhere in other members of the Gondwanan Onychophoran family Peripatopsidae [33,34,39]. In those other cases, allopatric and sometimes sympatric taxa are generally strongly and cleanly diverged from each other and with much lower genetic diversity and divergence within locations than we report here. Unlike South Africa and New Zealand, Tallaganda was periglaciated not glaciated, and a history of repeated habitat reorganization rather than eradication could explain the high diversity and complex local endemism seen in *E. rowelli* and other low-mobility inhabitants of Tallaganda [5]. Few areas within mainland Australia were glaciated, but one has been subject to molecular genetic study of an onychophoran, and supports these contentions: the entire genus *Planipapillus* that radiated in the alpine region of Australia has less mtDNA COI sequence divergence than seen in *E. rowelli* in a single log [72].

### Spatial Genetic Structuring: the Role of Geography

At Tallaganda, the broad association between the spatial distribution of genetic groups and catchment boundaries indicates a strong role for watersheds and associated environments in producing and / or maintain genetic structuring. However, this is apparently not the only causative factor, because some genetic breaks are spatially displaced from their putative geographic drivers. Genetic breaks that are spatially displaced from geographic features involved in population subdivision suggest a predominantly historical rather than contemporary role for those environmental elements in separating genetic groups [4,73]. A historical role is consistent with the effects of Pleistocene glacial cycling at Tallaganda, with saproxylic organisms likely to have contracted repeatedly to a series of wooded refugia separated by treeless steppe during treeline depression [11]. At broad geographic scales, Onychophora are generally found in regions where forests were not strongly affected by Pliocene/Pleistocene environments [74]. Contrary to this, our data suggest that *E. rowelli* has expanded into regions likely to have been treeless steppe during glacial cycling, presumably from nearby refugia.

The spatial locations of refugia where biodiversity may be generated or maintained through environmental change are important to consider for understanding and managing biodiversity [75,76]. The present data suggest at least two effective refugia sheltering Onychophora at Tallaganda through Pleistocene glacial cycling: one from which animals presently occupying HCR and ESR (groups 2 and 3) emerged on the most recent occasion after the Last Glacial Maximum (LGM, c. 20 Kya) [77], and one that was the source of *E. rowelli* presently occupying the rest of Tallaganda (group 1). Only a single major Pleistocene refuge was predicted at Tallaganda by Garrick et al. [4], hypothesised to be in ESR owing to it being deeply dissected and east-facing, thus most likely to have retained woody vegetation. This region was inferred as the major refuge for a Collembolon (subfamily Pseudachorutinae, gen. nov. sp. nov.) [12], and two species of flatworm [13], which are likely to have responded to climatic fluctuations in a similar way to *E. rowelli* [9]. In addition to the predicted refuge in ESR, we unexpectedly found evidence for a substantial refuge that could have harboured *E. rowelli* lineages in AR-PSR-BR. This implies that the conditions for survival of viable populations under glacial contraction were less stringent than originally imagined in Garrick et al. [4]. More detailed investigation of features, and spatial locations, of refuges in the Tallaganda model awaits more substantial data and coalescent and spatially-explicit analyses [75,78].

The hierarchical nature of the genetic structuring in the present data and the large number of groups implies a complicated evolutionary history [59]. Multi-staged histories of divergence have also been found in other saproxylic invertebrates at Tallaganda [13,16], and are likely the result of complex histories during the many climatic cycles in this region. One explanation for overlying recent and older structuring is the presence of ‘refugia within refugia’ [79,80]. Glaciation may result in contraction into refugia whose populations may not be panmictic. If expansion in different directions then occurs from different portions within refugia, fine-scale phylogeographic structure dating from the most recent glacial event will occur within the context of the larger, deeper structure [79,80]. The longer-term differentiation between broader refugial areas may not show the same patterns, because of the different processes and timescales involved. Our data are compatible with such a model.

The mtDNA phylogeny of *E. rowelli* revealed that there was little geographic structure in deep nodes but much more at the tips. This contrasts with patterns in other organisms at Tallaganda, whose clades are mostly monophyletic among catchments [4,13,16]. The exceptions are relatively good dispersers [7], and one taxon of funnel-web spider inferred to have radiated only recently within Tallaganda [17]. Three potential explanations for a phylogeny that is spatially structured only at the most recent timescales are: substantial historical admixture coupled with recent divergence in refugia followed by little admixture [81], incomplete lineage sorting [82], and limited resolution of the phylogeny owing to saturation at deeper divergences. These are not mutually exclusive. Contraction of diverged populations into relatively few refuges should result in admixture of mtDNA variation. Subsequent local expansion and drift (apparent in the nuclear data) would then produce the observed high degree of spatial sorting at tips. Next, *Euperipatoides rowelli* may experience incomplete lineage sorting of mtDNA owing to large effective population sizes. Although haplotypes should sort up to four times faster than nuclear markers in diploid species with equal operational sex ratios [82], *E. rowelli* mtDNA effective population size is likely to be inflated by female-biased demographics, low variance in female reproductive success, and high female philopatry, given rare patch extinction [23,26,28,29,83]. Finally, spatial signal might be lost deeper in the phylogeny if molecular saturation caused convergence among less-related sequences. This seems unlikely to offer much explanation for the main important pattern that the tree is geographically structured only at the tips, where any small amount of saturation would be well accounted for by the model of molecular evolution. Even at the level of genera, Gleeson et al. [84] found that mtDNA COI had some resolution.

### Barriers to gene flow

The patterns of genetic structuring produced during the proposed complex history of *E. rowelli* at Tallaganda are likely maintained and enhanced by low contemporary gene flow. Onychophoran biology renders them extremely susceptible to desiccation outside logs [26,27], so dispersal is risky. Even without postzygotic isolation, reproductive success after dispersal is likely to be lowered by features of the mating system and complex social biology including high aggression of females towards males from ‘foreign’ logs [23,25,28]. Gene flow between microgeographic regions should be low because the cooler, drier ridges between catchments are lower quality habitat housing fewer *E. rowelli* [26,85,86]. Finally, local adaptation and intra-genomic coadaptation within populations during isolation may inhibit gene flow through reduced hybrid fitness [87]. Evidence for reduced hybrid fitness has been observed between *E. rowelli* from different catchments at Tallaganda: ESR-AR hybrids have variation in leg number spanning 13-16 pairs and lateral asymmetries, whereas 15 pairs of legs is otherwise highly conserved in Australian Onychophora. This implies genomic incompatibilities between *E. rowelli* of neighbouring Tallaganda regions sufficient to affect fundamental body plans [22,24], consistent with some postzygotic reproductive isolation. However, apparently not all pairs of diagnosable catchment-based lineages of *E. rowelli* are subject to strong barriers to gene flow zones of contact: the two northernmost forms (HCR and ESR) appear to experience few adverse effects where they interbreed [88].

### Phenotypic character (colour pattern) incompletely mirrors neutral marker data

With one exception, colour pattern phenotype data were consistent with nuclear and mtDNA in identifying groups corresponding to the five *a priori* regions. The exception is that AR and PSR phenotypes are both ‘black’ with or without white spots yet the nuclear data indicate that black PSR and brightly spotted BR are more closely related to each other than either is to black AR. This implies that colour pattern phenotypes have been evolutionarily labile on less than the timescale taken to achieve drift-based differentiation of microsatellite loci. Scenarios include that the black pattern evolved independently in AR and PSR, that it swept faster than neutral markers from one form to the other, or that BR has secondarily acquired brightly-coloured spots. Any of these patterns might indicate a function for colouration [89], although it remains unclear what this could be. The differences among *E. rowelli* colour pattern forms seem very minor in regards to Crypsis; the species occupies the largely dark environment of rotting logs, and has poor vision including in the colour range of the characters in question [90,91].

### Closing comments and conservation management

Very fine-scale endemism was observed within *E. rowelli*, as with most other organisms studied at Tallaganda [5]. The exceptions are one of two funnel-web spiders and one of two skinks [7,17]. Overall, local endemism is likely in many saproxylic organisms at Tallaganda, highlighting the need for careful management of this and other topographically complex areas that are likely to be similarly rich in cryptic biodiversity [2,92].

## Supporting Information

File S1 **STRUCTURE microsatellite data file used, including location data (UTM grid 55H).**
(TXT)

File S2 **STRUCTURE main parameter file for analysis of File S1.**
(TXT)

Table S1 **Sampling locations for each data type.**
(DOCX)

Table S2 **Distribution of COI haplotypes by sampling location.**
(XLS)

Table S3 **Distribution of phenotypes by sampling location.**
(XLS)

Table S4 **Uncorrected pairwise differences between haplotypes.**
(XLS)

Table S5 **Microsatellite allele frequencies in nuclear genetic groups identified by STRUCTURE.**
(XLS)
