# Supplementary material for: Environmental Complexity and Biodiversity: The Multi-Layered Evolutionary History of a Log-Dwelling Velvet Worm in Montane Temperate Australia
Source: PLoS One. 2013 Dec 17;8(12):e84559. doi: 10.1371/journal.pone.0084559 (PMC3866147; doi:10.1371/journal.pone.0084559)
Supplement: Table S1 — Sampling locations for each data type. (DOCX) [file pone.0084559.s003.docx]

**Table S1. Sampling locations for each data type.** Locations of decaying logs from which *Euperipatoides rowelli* were sampled for this study, with the number of individuals that were scored for each of the three data types in each log. Site numbers define the groups for each data type used in statistical analyses; where bold, site contains multiple logs for that data type. All eastings and northings are in grid 55H. Catchments are as defined in Sunnucks P, Blacket MJ, Taylor JM, Sands CJ, Ciavaglia SA, et al. (2006) A tale of two flatties: different responses of two terrestrial flatworms to past environmental climatic fluctuations at Tallaganda in montane southeastern Australia. Molecular Ecology 15: 4513-4531.

|  |  |  |  |  | **Microsatellite** | | **Mitochondrial** | | **Colouration** | |
| --- | --- | --- | --- | --- | --- | --- | --- | --- | --- | --- |
| **#** | **Log** | **Easting** | **Northing** | **Catchment** | **Site** | **n** | **Site** | **n** | **Site** | **n** |
| 1 | B17 | 726629 | 5988396 | BR | 1 | 6 | - | - | 1 | 3 |
| 2 | B22 | 729528 | 5991637 | BR | - | - | - | - | **2** | 8 |
| 3 | B21 | 729531 | 5991665 | BR | 2 | 8 | - | - | **2** | 9 |
| 4 | D34 | 725806 | 5991858 | BR | - | - | 1 | 7 | - | - |
| 5 | B20 | 726529 | 5991939 | BR | - | - | - | - | 3 | 1 |
| 6 | B61 | 727858 | 5992500 | BR | - | - | - | - | 4 | 5 |
| 7 | Z433 | 725578 | 5993181 | BR | - | - | 2 | 1 | - | - |
| 8 | Z434 | 725611 | 5993191 | BR | - | - | 3 | 2 | - | - |
| 9 | Z439 | 725687 | 5993221 | BR | - | - | 4 | 2 | - | - |
| 10 | B23 | 725560 | 5993230 | BR | - | - | - | - | 5 | 6 |
| 11 | B10 | 728645 | 5993359 | BR | - | - | - | - | **6** | 1 |
| 12 | D26 | 723269 | 5993466 | BR | - | - | 5 | 31 | - | - |
| 13 | B11 | 728691 | 5993480 | BR | - | - | - | - | **6** | 8 |
| 14 | OB62 | 727751 | 5993605 | BR | - | - | - | - | 7 | 8 |
| 15 | B25 | 724199 | 5993833 | NU | - | - | - | - | 8 | 16 |
| 16 | B01 | 726700 | 5993900 | BR | **3** | 4 | - | - | - | - |
| 17 | B03 | 726700 | 5993900 | BR | **3** | 8 | - | - | - | - |
| 18 | B02 | 726700 | 5994000 | BR | **3** | 7 | - | - | - | - |
| 19 | B24 | 725009 | 5994296 | NU | - | - | - | - | 9 | 1 |
| 20 | B64 | 725199 | 5995252 | NU | - | - | - | - | **10** | 4 |
| 21 | Z437 | 725719 | 5995520 | NU | 4 | 4 | - | - | - | - |
| 22 | B34 | 725669 | 5995528 | NU | - | - | - | - | **10** | 2 |
| 23 | B65 | 725595 | 5995920 | NU | - | - | - | - | **11** | 5 |
| 24 | B66 | 725755 | 5995935 | NU | 5 | 6 | - | - | **11** | 5 |
| 25 | B67 | 725767 | 5995963 | NU | - | - | - | - | **11** | 1 |
| 26 | B14 | 728625 | 5996050 | BR | - | - | - | - | 12 | 9 |
| 27 | B12 | 728699 | 5997674 | BR | - | - | - | - | 13 | 4 |
| 28 | B33 | 726474 | 5997718 | BR | - | - | - | - | 14 | 1 |
| 29 | D25 | 727008 | 5999199 | BR | - | - | 6 | 5 | - | - |
| 30 | B71 | 733843 | 5999788 | BR | **6** | 2 | - | - | 15 | 2 |
| 31 | B70 | 733852 | 5999815 | BR | **6** | 3 | - | - | 16 | 3 |
| 32 | B72 | 732504 | 6000121 | BR | - | - | - | - | 17 | 7 |
| 33 | B32 | 728702 | 6000603 | BR | - | - | - | - | 18 | 3 |
| 34 | B73 | 730662 | 6000734 | BR | - | - | - | - | 19 | 1 |
| 35 | B74 | 728911 | 6000942 | NU | - | - | - | - | 20 | 2 |
| 36 | B29 | 732751 | 6001903 | BR | **7** | 5 | - | - | **21** | 5 |
| 37 | B28 | 732771 | 6001915 | BR | **7** | 5 | - | - | **21** | 4 |
| 38 | B83 | 726252 | 6002800 | NU | - | - | - | - | 22 | 23 |
| 39 | B27 | 730734 | 6003286 | NU | - | - | - | - | 23 | 9 |
| 40 | D20 | 732032 | 6003390 | BR | 8 | 10 | 7 | 11 | - | - |
| 41 | D21 | 732277 | 6004142 | BR | **9** | 3 | 8 | 3 | - | - |
| 42 | D22 | 732297 | 6004208 | BR | **9** | 10 | 9 | 16 | - | - |
| 43 | D23 | 732310 | 6004758 | BR | 10 | 10 | 10 | 20 | - | - |
| 44 | D24 | 732518 | 6005152 | BR | - | - | 11 | 4 | - | - |
| 45 | D19 | 732535 | 6005404 | BR | 11 | 9 | 12 | 7 | - | - |
| 46 | B75 | 732222 | 6005808 | BR | - | - | - | - | 24 | 1 |
| 47 | OPC | 728852 | 6005865 | NU | - | - | - | - | 25 | 11 |
| 48 | B76 | 732223 | 6005866 | BR | - | - | - | - | **26** | 10 |
| 49 | OB76 | 732223 | 6005866 | BR | - | - | - | - | **26** | 1 |
| 50 | B77 | 732235 | 6005887 | BR | - | - | - | - | **26** | 6 |
| 51 | B35 | 731797 | 6006801 | BR | - | - | - | - | 27 | 6 |
| 52 | B78 | 731779 | 6007350 | BR | - | - | - | - | 28 | 9 |
| 53 | OB78 | 731779 | 6007350 | BR | - | - | - | - | 28 | 3 |
| 54 | Z426 | 731874 | 6007469 | BR | 12 | 10 | - | - | - | - |
| 55 | B82 | 730318 | 6007481 | NU | - | - | - | - | 29 | 2 |
| 56 | PEPP | 728650 | 6007900 | NU | - | - | - | - | 30 | 3 |
| 57 | B79 | 731538 | 6008349 | NU | 13 | 10 | - | - | 31 | 17 |
| 58 | B81 | 731502 | 6009609 | BR | - | - | - | - | **32** | 5 |
| 59 | B80 | 731439 | 6009616 | BR | - | - | - | - | **32** | 9 |
| 60 | BFR | 728100 | 6010100 | NU | - | - | - | - | 33 | 1 |
| 61 | D16 | 731465 | 6010428 | NU | 14 | 10 | 13 | 10 | - | - |
| 62 | D17 | 731416 | 6010711 | NU | - | - | 14 | 5 | - | - |
| 63 | D18 | 731421 | 6010725 | NU | - | - | 15 | 2 | - | - |
| 64 | B87 | 725956 | 6011246 | NU | - | - | - | - | **34** | 34 |
| 65 | OB87 | 725956 | 6011246 | NU | - | - | - | - | **34** | 4 |
| 66 | B86 | 725934 | 6011288 | NU | - | - | - | - | **34** | 11 |
| 67 | B84 | 727624 | 6011672 | NU | - | - | - | - | **35** | 1 |
| 68 | B85 | 727624 | 6011672 | NU | - | - | - | - | **35** | 7 |
| 69 | OB84 | 727624 | 6011672 | NU | - | - | - | - | **35** | 1 |
| 70 | B88 | 724618 | 6012891 | NU | - | - | - | - | 36 | 13 |
| 71 | L08 | 730000 | 6014250 | PSR | - | - | 16 | 4 | - | - |
| 72 | L10 | 729990 | 6014307 | PSR | - | - | **17** | 10 | - | - |
| 73 | L12 | 729990 | 6014307 | PSR | 15 | 1 | **17** | 5 | - | - |
| 74 | BFT2 | 731100 | 6014400 | PSR | - | - | - | - | **37** | 12 |
| 75 | L01 | 730000 | 6014400 | PSR | - | - | **18** | 11 | - | - |
| 76 | L02 | 730000 | 6014400 | PSR | 16 | 11 | **18** | 2 | - | - |
| 77 | L03 | 730150 | 6014400 | PSR | 17 | 9 | - | - | - | - |
| 78 | L04 | 730150 | 6014400 | PSR | - | - | 19 | 16 | - | - |
| 79 | L05 | 730250 | 6014400 | PSR | - | - | **20** | 3 | - | - |
| 80 | L06 | 730250 | 6014400 | PSR | 18 | 9 | **20** | 12 | - | - |
| 81 | L30 | 730200 | 6014400 | PSR | 19 | 10 | - | - | - | - |
| 82 | BFT | 731150 | 6014550 | PSR | - | - | - | - | **37** | 5 |
| 83 | C14 | 739034 | 6014762 | PSR | - | - | - | - | **38** | 7 |
| 84 | C13 | 739032 | 6014764 | PSR | - | - | - | - | **38** | 7 |
| 85 | L13 | 730900 | 6014800 | PSR | - | - | 21 | 2 | - | - |
| 86 | C01 | 727060 | 6015142 | NU | - | - | - | - | **39** | 4 |
| 87 | C01a | 727060 | 6015142 | NU | - | - | - | - | **39** | 9 |
| 88 | C02 | 727068 | 6015157 | NU | - | - | - | - | **39** | 1 |
| 89 | L17 | 727300 | 6015400 | PSR | 21 | 10 | 22 | 17 | - | - |
| 90 | T11 | 727150 | 6015400 | NU | 20 | 5 | - | - | - | - |
| 91 | B89 | 726156 | 6015478 | NU | - | - | - | - | 40 | 1 |
| 92 | C12 | 736150 | 6015726 | PSR | 22 | 8 | - | - | 41 | 4 |
| 93 | C04 | 727073 | 6016735 | PSR | - | - | - | - | **42** | 2 |
| 94 | C05 | 727064 | 6016741 | PSR | - | - | - | - | **42** | 7 |
| 95 | B99 | 732557 | 6016988 | PSR | 23 | 10 | - | - | **43** | 11 |
| 96 | B98 | 732532 | 6017000 | PSR | - | - | - | - | **43** | 33 |
| 97 | B92 | 727900 | 6017107 | PSR | - | - | - | - | 44 | 4 |
| 98 | C11 | 734869 | 6017391 | PSR | - | - | - | - | 45 | 2 |
| 99 | C15 | 741696 | 6017393 | PSR | - | - | - | - | **46** | 22 |
| 100 | OC15 | 741696 | 6017393 | PSR | - | - | - | - | **46** | 6 |
| 101 | B90 | 728675 | 6017661 | PSR | - | - | - | - | **47** | 1 |
| 102 | OB90 | 728675 | 6017673 | PSR | - | - | - | - | **47** | 2 |
| 103 | B97 | 732638 | 6018172 | PSR | - | - | - | - | 48 | 8 |
| 104 | C10 | 734762 | 6018234 | PSR | 24 | 10 | - | - | 49 | 16 |
| 105 | B94 | 731205 | 6018981 | PSR | - | - | - | - | 50 | 8 |
| 106 | B95 | 731188 | 6018986 | PSR | - | - | - | - | 51 | 3 |
| 107 | T13 | 730900 | 6021000 | PSR | 25 | 5 | - | - | - | - |
| 108 | T14 | 730900 | 6023300 | PSR | 26 | 10 | - | - | - | - |
| 109 | C16 | 731162 | 6024105 | PSR | - | - | - | - | 52 | 6 |
| 110 | C17 | 731002 | 6024588 | PSR | - | - | - | - | 53 | 12 |
| 111 | C08 | 726169 | 6024696 | ESR-Je | - | - | - | - | 54 | 7 |
| 112 | OC08 | 726169 | 6024696 | ESR-Je | - | - | - | - | 55 | 6 |
| 113 | D08 | 731644 | 6025308 | PSR | 27 | 6 | - | - | - | - |
| 114 | J06 | 725688 | 6025341 | ESR-Je | - | - | - | - | **56** | 7 |
| 115 | J05 | 725685 | 6025408 | ESR-Je | - | - | - | - | **56** | 12 |
| 116 | L15 | 727400 | 6027600 | ESR-Je | 28 | 10 | 23 | 17 | - | - |
| 117 | B39 | 727444 | 6027676 | ESR-Je | - | - | - | - | 57 | 20 |
| 118 | T01 | 726600 | 6027900 | ESR-Je | **29** | 3 | - | - | - | - |
| 119 | T02 | 725600 | 6028100 | ESR-Je | **29** | 5 | - | - | - | - |
| 120 | B38 | 724810 | 6028592 | ESR-Je | - | - | - | - | 58 | 4 |
| 121 | W08 | 729856 | 6029439 | ESR-Je | - | - | - | - | 59 | 3 |
| 122 | D02 | 729830 | 6029709 | ESR-Je | 30 | 10 | - | - | - | - |
| 123 | A04 | 724600 | 6030100 | ESR-Je | 31 | 10 | - | - | - | - |
| 124 | B40 | 724513 | 6030393 | ESR-Je | - | - | - | - | 60 | 1 |
| 125 | OW07 | 729696 | 6030477 | ESR-Je | - | - | - | - | **61** | 1 |
| 126 | W07 | 729696 | 6030477 | ESR-Je | - | - | - | - | **61** | 2 |
| 127 | A01 | 724200 | 6030500 | AR | 32 | 10 | - | - | - | - |
| 128 | Z422 | 724168 | 6030775 | AR | - | - | 24 | 5 | - | - |
| 129 | B36 | 723992 | 6030822 | AR | - | - | - | - | 62 | 3 |
| 130 | C18 | 725373 | 6031813 | AR | 33 | 10 | - | - | **63** | 11 |
| 131 | OC18 | 725373 | 6031813 | AR | - | - | - | - | **63** | 6 |
| 132 | R05 | 722950 | 6032800 | AR | 34 | 9 | - | - | - | - |
| 133 | D03 | 728877 | 6032802 | ESR-Je | 35 | 6 | - | - | - | - |
| 134 | D04 | 727864 | 6033766 | ESR-Je | 36 | 10 | - | - | - | - |
| 135 | T06 | 725500 | 6033800 | ESR-Je | 37 | 1 | - | - | - | - |
| 136 | C19 | 726742 | 6033885 | ESR-Je | - | - | - | - | 64 | 12 |
| 137 | H31 | 727636 | 6035065 | ESR-Je | **38** | 4 | - | - | - | - |
| 138 | H32 | 727636 | 6035065 | ESR-Je | **38** | 6 | - | - | - | - |
| 139 | C20 | 726464 | 6036302 | AR | - | - | - | - | 65 | 14 |
| 140 | B57 | 723821 | 6038704 | AR | - | - | - | - | 66 | 5 |
| 141 | C21 | 727520 | 6039756 | AR | - | - | - | - | 67 | 24 |
| 142 | T100 | 729789 | 6040942 | ESR-Je | - | - | - | - | 68 | 7 |
| 143 | R09 | 723400 | 6041300 | AR | 39 | 2 | - | - | - | - |
| 144 | W05 | 729733 | 6041910 | ESR-Je | - | - | - | - | 69 | 7 |
| 145 | E17 | 729100 | 6043200 | ESR-Je | - | - | 25 | 15 | - | - |
| 146 | H08 | 729448 | 6044511 | ESR-Je | 40 | 10 | - | - | - | - |
| 147 | H03 | 730700 | 6044900 | ESR-Je | 41 | 10 | - | - | - | - |
| 148 | H48 | 730113 | 6045159 | ESR-Je | 42 | 10 | - | - | - | - |
| 149 | H30 | 729575 | 6045932 | ESR-Je | 43 | 8 | - | - | - | - |
| 150 | H54 | 729508 | 6046840 | ESR-Je | 44 | 10 | - | - | - | - |
| 151 | H15 | 726900 | 6047250 | AR | 45 | 4 | - | - | - | - |
| 152 | SH06 | 729470 | 6047359 | ESR-Ba | - | - | - | - | **70** | 21 |
| 153 | SH07 | 729457 | 6047359 | ESR-Ba | - | - | - | - | **70** | 12 |
| 154 | SH05 | 729453 | 6047421 | ESR-Ba | - | - | - | - | **70** | 19 |
| 155 | H06 | 728612 | 6047616 | ESR-Ba | 46 | 10 | - | - | - | - |
| 156 | H26 | 729636 | 6047697 | ESR-Ba | 47 | 10 | - | - | - | - |
| 157 | H14 | 726800 | 6048450 | AR | 48 | 16 | - | - | - | - |
| 158 | H22 | 728758 | 6049358 | ESR-Ba | 49 | 10 | 26 | 7 | - | - |
| 159 | E14 | 728749 | 6049598 | ESR-Ba | - | - | 27 | 20 | - | - |
| 160 | SH08 | 728961 | 6049621 | ESR-Ba | - | - | 28 | 6 | **71** | 21 |
| 161 | SH09 | 728909 | 6049656 | ESR-Ba | - | - | - | - | **71** | 12 |
| 162 | H13 | 727400 | 6050150 | ESR-Ba | 50 | 2 | - | - | - | - |
| 163 | N109 | 728796 | 6050849 | ESR-Ba | - | - | 29 | 6 | - | - |
| 164 | N108 | 728696 | 6050883 | ESR-Ba | - | - | **30** | 1 | - | - |
| 165 | E12 | 729138 | 6051235 | ESR-Ba | 51 | 9 | **30** | 10 | - | - |
| 166 | E13 | 729138 | 6051235 | ESR-Ba | - | - | 31 | 10 | - | - |
| 167 | H12 | 727250 | 6051500 | ML | 52 | 10 | - | - | - | - |
| 168 | J17 | 727232 | 6051641 | ESR-Ba | - | - | - | - | 72 | 2 |
| 169 | H16 | 727200 | 6051800 | ML | **53** | 2 | - | - | - | - |
| 170 | H17 | 727200 | 6051800 | ML | **53** | 5 | - | - | - | - |
| 171 | R02 | 727500 | 6052100 | ESR-Ba | - | - | - | - | **73** | 1 |
| 172 | R03 | 727500 | 6052100 | ESR-Ba | - | - | - | - | **73** | 1 |
| 173 | H20 | 729146 | 6052105 | ESR-Ba | 54 | 9 | - | - | - | - |
| 174 | SH02 | 727934 | 6053050 | ESR-Ba | - | - | - | - | **74** | 26 |
| 175 | SH01 | 727953 | 6053064 | ESR-Ba | - | - | 32 | 3 | **74** | 29 |
| 176 | SH03 | 728009 | 6053077 | ESR-Ba | - | - | - | - | **74** | 6 |
| 177 | SH04 | 728063 | 6053116 | ESR-Ba | - | - | - | - | **74** | 28 |
| 178 | E01 | 728211 | 6053368 | ESR-Ba | 55 | 10 | 33 | 19 | - | - |
| 179 | D05 | 727909 | 6053519 | ESR-Ba | - | - | - | - | 75 | 1 |
| 180 | VM01 | 726479 | 6053627 | ESR-Ba | - | - | - | - | **76** | 1 |
| 181 | VM03 | 726642 | 6053665 | ESR-Ba | - | - | - | - | **76** | 1 |
| 182 | E02 | 728344 | 6053827 | ESR-Ba | - | - | 34 | 15 | - | - |
| 183 | SH10 | 728265 | 6053964 | ESR-Ba | 56 | 10 | 35 | 4 | 77 | 22 |
| 184 | W01 | 727809 | 6054072 | ESR-Ba | - | - | - | - | **78** | 13 |
| 185 | W03 | 727828 | 6054113 | ESR-Ba | - | - | - | - | **78** | 5 |
| 186 | W04 | 727809 | 6054118 | ESR-Ba | - | - | - | - | **78** | 18 |
| 187 | W02 | 727937 | 6054149 | ESR-Ba | - | - | - | - | **78** | 4 |
| 188 | E03 | 728479 | 6054379 | ESR-Ba | 57 | 10 | **36** | 19 | - | - |
| 189 | E05 | 728479 | 6054379 | ESR-Ba | 58 | 10 | **36** | 16 | - | - |
| 190 | E06 | 728479 | 6054379 | ESR-Ba | - | - | **36** | 17 | - | - |
| 191 | E07 | 728479 | 6054379 | ESR-Ba | - | - | **36** | 16 | - | - |
| 192 | E08 | 728479 | 6054379 | ESR-Ba | - | - | **36** | 17 | - | - |
| 193 | E09 | 728479 | 6054379 | ESR-Ba | - | - | **36** | 11 | - | - |
| 194 | Z410 | 728039 | 6054860 | ESR-Ba | 59 | 9 | 37 | 8 | - | - |
| 195 | N42 | 726397 | 6055176 | ESR-Ba | - | - | 38 | 3 | - | - |
| 196 | VM08 | 726403 | 6056075 | ESR-Ba | - | - | 39 | 2 | - | - |
| 197 | J08 | 726623 | 6057977 | ML | - | - | 40 | 5 | - | - |
| 198 | J11 | 726845 | 6058523 | ML | - | - | 41 | 3 | - | - |
| 199 | J12 | 726849 | 6059166 | ML | - | - | 42 | 3 | - | - |
| 200 | N41 | 726792 | 6059325 | ML | - | - | 43 | 1 | - | - |
| 201 | C26 | 726997 | 6061253 | ML | - | - | 44 | 12 | - | - |
| 202 | J21 | 728104 | 6063430 | ML | - | - | 45 | 4 | - | - |
| 203 | C27 | 727831 | 6064434 | ML | - | - | 46 | 20 | - | - |
| 204 | N106 | 731624 | 6065733 | ESR-Ba | 60 | 7 | 47 | 7 | - | - |
| 205 | Z409 | 732843 | 6065998 | ESR-Ba | 61 | 11 | 48 | 2 | - | - |
| 206 | N34 | 734500 | 6066500 | ESR-Ba | 62 | 9 | 49 | 20 | - | - |
| 207 | N35 | 734500 | 6066500 | ESR-Ba | - | - | 49 | 7 | - | - |
| 208 | C36 | 729549 | 6066742 | ESR-Ba | 63 | 10 | 50 | 10 | 79 | 7 |
| 209 | N107 | 734071 | 6066779 | ESR-Ba | 64 | 5 | 51 | 3 | - | - |
| 210 | N40 | 727854 | 6067476 | ML | - | - | 52 | 14 | - | - |
| 211 | N01 | 730010 | 6067600 | ML | **65** | 5 | - | - | - | - |
| 212 | N02 | 730010 | 6067600 | ML | **65** | 10 | - | - | - | - |
| 213 | J22 | 737614 | 6068214 | ESR-Ba | - | - | - | - | **80** | 1 |
| 214 | J23 | 737566 | 6068244 | ESR-Ba | - | - | - | - | **80** | 1 |
| 215 | C24 | 730660 | 6068381 | ML | 66 | 9 | - | - | **81** | 40 |
| 216 | OC24 | 730660 | 6068381 | ML | - | - | - | - | **81** | 3 |
| 217 | J29 | 733874 | 6070844 | HCR | - | - | - | - | 82 | 3 |
| 218 | C33 | 738497 | 6071151 | HCR | 67 | 10 | - | - | 83 | 23 |
| 219 | J26 | 732380 | 6071780 | HCR | 68 | 3 | - | - | 84 | 3 |
| 220 | J27 | 733706 | 6072224 | HCR | - | - | - | - | 85 | 2 |
| 221 | C32 | 738413 | 6072888 | HCR | 69 | 10 | - | - | 86 | 14 |
| 222 | C38 | 731305 | 6074312 | HCR | - | - | - | - | 87 | 6 |
| 223 | OC41 | 730870 | 6074422 | HCR | - | - | - | - | 88 | 2 |
| 224 | Z405 | 731485 | 6074429 | HCR | **70** | 1 | - | - | - | - |
| 225 | Z406 | 731505 | 6074453 | HCR | **70** | 1 | - | - | - | - |
| 226 | N104 | 731522 | 6074462 | HCR | - | - | 53 | 8 | - | - |
| 227 | Z407 | 731414 | 6074490 | HCR | 70 | 7 | 54 | 3 | - | - |
| 228 | C40 | 730600 | 6074800 | HCR | 71 | 4 | - | - | **88** | 3 |
| 229 | OC39 | 730600 | 6074800 | HCR | - | - | - | - | **88** | 4 |
| 230 | N05 | 731462 | 6075081 | HCR | - | - | 55 | 10 | - | - |
| 231 | N30 | 733334 | 6075216 | HCR | **72** | 1 | 56 | 10 | - | - |
| 232 | N03 | 733334 | 6075217 | HCR | **72** | 7 | - | - | - | - |
| 233 | N04 | 733334 | 6075217 | HCR | **72** | 3 | - | - | - | - |
| 234 | C29 | 738293 | 6076446 | HCR | 73 | 9 | - | - | 89 | 5 |
| 235 | J25 | 730536 | 6076675 | HCR | - | - | - | - | 90 | 3 |
| 236 | C28 | 739186 | 6076927 | HCR | - | - | - | - | 91 | 9 |
| 237 | N32 | 736100 | 6077500 | HCR | - | - | 57 | 4 | - | - |
| 238 | C42 | 731714 | 6082735 | HCR | - | - | - | - | 92 | 2 |
